# Forced swim test induces divergent global transcriptomic alterations in the hippocampus of high versus low novelty-seeker rats

**DOI:** 10.1186/1479-7364-8-4

**Published:** 2014-02-25

**Authors:** Pothitos M Pitychoutis, Despina Sanoudou, Margarita Papandreou, Dimitris Nasias, Marianna Kouskou, Craig R Tomlinson, Panagiotis A Tsonis, Zeta Papadopoulou-Daifoti

**Affiliations:** 1 Department of Biology & Center for Tissue Regeneration and Engineering (TREND), University of Dayton, 300 College Park, Dayton, OH 45469-2320, USA; 2 Department of Pharmacology, Medical School, National & Kapodistrian University of Athens, 75 Mikras Asias StreetGoudi, Athens 11527, Greece; 3 Department of Medicine, Dartmouth Hitchcock Medical Center, Dartmouth College, Lebanon, NH 03756, USA; 4 Department of Pharmacology and Toxicology, Dartmouth Hitchcock Medical Center, Dartmouth College, Lebanon, NH 03756, USA

**Keywords:** Stress, Gene expression, Rats, Depression, Individual differences, Hippocampus, FST, Neurogenesis, Neuroplasticity, Apoptosis

## Abstract

**Background:**

Many neuropsychiatric disorders, including stress-related mood disorders, are complex multi-parametric syndromes. Susceptibility to stress and depression is individually different. The best animal model of individual differences that can be used to study the neurobiology of affect regards spontaneous reactions to novelty. Experimentally, when naive rats are exposed to the stress of a novel environment, they display a highly variable exploratory activity and are classified as high or low responders (HR or LR, respectively). Importantly, HR and LR rats do not seem to exhibit a substantial differentiation in relation to their ‘depressive-like’ *status* in the forced swim test (FST), a widely used animal model of ‘behavioral despair’. In the present study, we investigated whether FST exposure would be accompanied by phenotype-dependent differences in hippocampal gene expression in HR and LR rats.

**Results:**

HR and LR rats present a distinct behavioral pattern in the pre-test session but develop comparable depressive-like status in the second FST session. At 24 h following the second FST session, HR and LR rats (stressed and unstressed controls) were sacrificed and hippocampal samples were independently analyzed on whole rat genome Illumina arrays. Functional analysis into pathways and networks was performed using Ingenuity Pathway Analysis (IPA) software. Notably, hippocampal gene expression signatures between HR and LR rats were markedly divergent, despite their comparable depressive-like status in the FST. These molecular differences are reflected in both the extent of transcriptional remodeling (number of significantly changed genes) and the types of molecular pathways affected following FST exposure. A markedly higher number of genes (i.e., 2.28-fold) were statistically significantly changed following FST in LR rats, as compared to their HR counterparts. Notably, genes associated with neurogenesis and synaptic plasticity were induced in the hippocampus of LR rats in response to FST, whereas in HR rats, FST induced pathways directly or indirectly associated with induction of apoptotic mechanisms.

**Conclusions:**

The markedly divergent gene expression signatures exposed herein support the notion that the hippocampus of HR and LR rats undergoes distinct transcriptional remodeling in response to the same stress regimen, thus yielding a different FST-related ‘endophenotype’, despite the seemingly similar depressive-like phenotype.

## Background

Many neuropsychiatric disorders, including stress-related mood disorders, are complex multi-parametric syndromes. Strikingly, susceptibility to depression and stress differs between individuals. Accurate diagnosis is hard to establish and current pharmacotherapeutic strategies suffer from significant variability in effectiveness, making the understanding of inter-individual variations crucial to unveiling effective new treatments [1].

The best animal model of individual differences that can be used to study the neurobiology of affect regards spontaneous reactions to novelty [2]. Experimentally, when naive rats are exposed to the stress of a novel environment, they display a highly variable exploratory activity; some rats are characterized by high rates of locomotor reactivity (high responders (HR)) whereas others by low rates (low responders (LR)) (for a detailed review on the HR/LR model, the reader is referred to [2,3]). Novelty-seeking is an affect-related trait that has been associated with anxiety and emotional reactivity [4] as well as with depressive symptomatology [5,6] in humans and in rodents.

The forced swim test (FST) [7] is a widely implemented animal model of behavioral despair with high predictive validity for agents with antidepressive potential [8,9]. Increased passive behavioral responses in FST, such as immobility, and decreased active behaviors like swimming or climbing are thought to be a clear indication of ‘depressive-like’ symptomatology [10,11]. Interestingly, male HR and LR rats do not exhibit a substantial differentiation in relation to their depressive-like *status* in the FST [4,12,13]. However, previous results from our lab indicate that HR and LR rats may exhibit a different behavioral pattern characterized by distinct active behavioral reactions expressed during either the first or the second exposure to water [4]. Moreover, we recently reported that chronic antidepressant treatment with clomipramine may attenuate depressive-like symptomatology in FST in male HR but not in their LR counterparts [12]. This evidence combined suggests that idiosyncratic responses to novelty may lead to a phenotypically similar depressive-like outcome, through different, yet unknown, molecular mechanisms.

In search of the brain structures likely to be involved in these processes, the hippocampus stands out due to its central role in the detection of novelty and the guidance of behavioral responses on the basis of familiarity [14]. Furthermore, the hippocampus has been the focus of multiple studies on the pathophysiology and treatment of stress-related disorders [15-17]. Indeed, stress often has detrimental effects on the integrity and function of the hippocampus. These effects include alterations in the activity of monoaminergic systems, in dendritic and synaptic remodeling, as well as in levels of adult hippocampal neurogenesis [18].

In the present study, we investigated whether FST exposure would be accompanied by phenotype-dependent differences in hippocampal gene expression in HR and LR rats. The markedly divergent gene expression signatures exposed herein support the notion that the hippocampus of HR and LR rats undergoes distinct transcriptional remodeling in response to the same stress regimen, thus yielding a different FST-related ‘endophenotype’ , despite the seemingly similar depressive-like phenotype.

## Results

### HR and LR rats present with distinct behavioral pattern in the pre-test session but comparable depressive-like status in the second FST session

**Figure 1:**
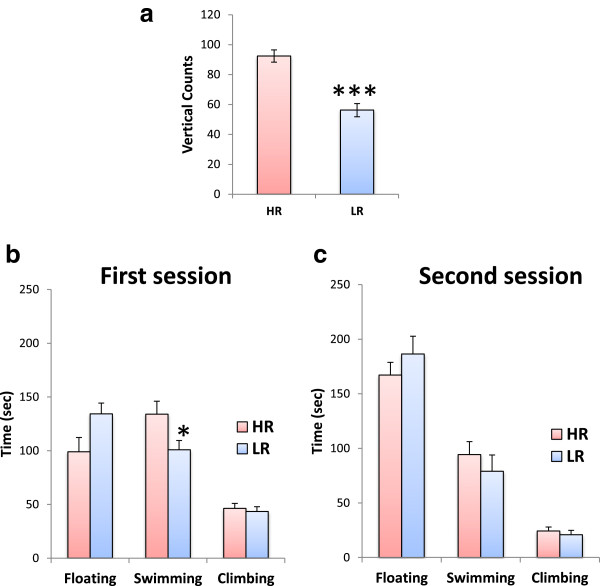
**Behavioral data. (a)** HR/LR classification: the initial classification of rats into HR or LR phenotype on the basis of their vertical locomotor activity (i.e., vertical counts) in an open field (*N* = 12 per phenotype) was assessed with one-way ANOVAs. The structure of HR and LR rats' behavior (i.e., immobility, swimming, climbing) (*N* = 7 per group) in the **(b)** first (pre-test) and **(c)** second (test) FST sessions was assessed with separate one-way ANOVAs. *Bars* represent means ± SEM; *p < 0.05; ***p < 0.001.

A one-way analysis of variance (ANOVA) showed that HR rats are characterized by higher vertical activity as compared to their LR counterparts (*F*_(1,23)_ = 36.234; *p* < 0.001; Figure 1a). This level of significance was maintained when these initial groups were dichotomized in order for the control and FST groups to be formed (data not shown). Most importantly, during the pre-test FST session, a one-way ANOVA showed that LR rats spent less time swimming around the cylinder, as compared to their HR counterparts (*F*_(1,13)_ = 4.957; *p* = 0.046; Figure 1b). In LR rats, this decrease in swimming was attributed to a non-significant increase of immobility. The same analysis showed that the structure of HR and LR rats' behavior was similar during the second FST session (Figure 1c). Specifically, both HR and LR rats exhibited increased floating behavior and decreased active behaviors (i.e., climbing and swimming) during the second session, which reflect the establishment of depressive-like behavior in this test.

### Hippocampal gene expression signature changes in HR and LR rats following exposure to FST

**Figure 2:**
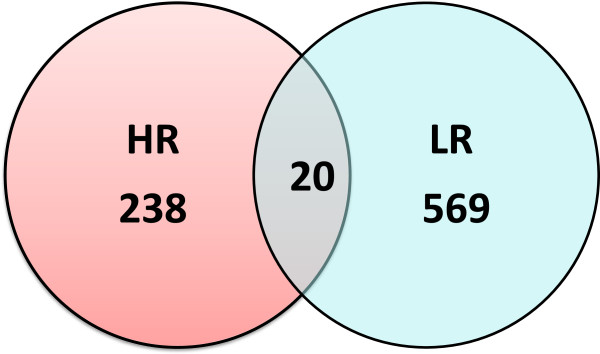
**Exposure to FST affected a different number of transcripts in HR versus LR rats (238 versus 569).** Notably, only 20 common transcripts were affected in both novelty-seeking phenotypes.

The global gene expression signatures in the hippocampus of HR and LR rats were assessed in rats exposed to FST versus their respective unstressed counterparts. Bioinformatic analysis revealed significant changes in 258 transcripts (243 upregulated; 15 downregulated) in HR rats upon FST exposure (Additional files 1 and 2). On the other hand, in LR rats, 589 transcripts were statistically significantly changed, all of which were upregulated. Despite the large number of significant changes, only 20 transcripts were affected in both novelty-seeking phenotypes (Figure 2; Additional file 3).

### Gene Ontology (GO) classification

To determine the biological processes and molecular functions likely to be affected by the significant gene expression changes, a comparative analysis tool provided by the ‘GeneCodis3’ software was used for the Gene Ontology classification of the differentially expressed genes in both groups. GO level 5 annotations were obtained for 117 unique genes in HR rats and 354 unique genes in LR rats (only 9 genes were common in both groups). Focusing on the ‘biological process’ (BP) of the unique genes, 50 processes (annotations) in HR and 189 processes in LR rats were found to be statistically significantly changed. In HR rats, the BPs most highly affected were the ‘cellular macromolecule metabolic process’ (56 genes) and the ‘cellular biosynthetic process’ (42 genes) whereas in LR rats, the ‘regulation of cellular process’ (154 genes) and ‘cellular macromolecule metabolic process’ (125 genes). As far as the 9 common genes are concerned, no annotations were significantly enriched.

**Figure 3:**
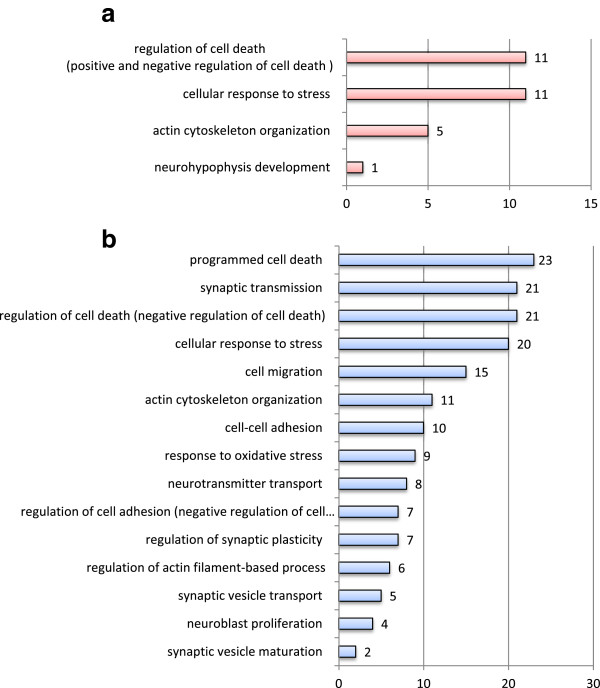
**Distribution of differentially expressed genes according to GO BP in (a) HR and (b) LR rats.** GeneCodis functional GO BP level 5 categories that meet conservative statistical criteria (hypergeometric distribution and false discovery rate (FDR) test, *p* < 0.05) and include any one of the selected relevant keywords (i.e., neuro-, synaptic, stress, plasticity, death, cytoskeleton, migration, and adhesion). *Numbers* in the chart denote the number of genes grouped in each annotation.

In order to depict those processes orchestrating possible hippocampal structural and/or functional alterations in response to FST in the two novelty-seeking phenotypes, an additional set of selection criteria was applied, depicting statistically significant GO categories selected by one or more of the following keywords: neuro-, synaptic, stress, plasticity, cell death, cytoskeleton, migration, and adhesion (Figure 3). This approach unveiled even greater differences between the molecular response of HR and LR rats to FST exposure. Notably, ‘synaptic transmission’ (21 genes), ‘cell migration’ (15 genes), ‘cell-cell adhesion’ (10 genes), ‘neurotransmitter transport’ (8 genes), and ‘neuroblast proliferation’ (4 genes) are among the GO BPs found to be represented only in the LR group, while ‘regulation of cell death’ (11 HR versus 21 LR genes), ‘cellular response to stress’ (11 HR versus 20 LR genes), and ‘actin cytoskeleton organization’ (5 HR versus 11 LR genes) were found to be affected in both groups.

### Ingenuity Pathway Analysis (IPA)

The GO findings were confirmed and further enriched through IPA. In HR rats, IPA analysis indicated that the observed gene expression changes were associated with 73 statistically significant functional categories including ‘cellular growth and proliferation’ (45 genes), ‘cellular development’ (24 genes), ‘cellular movement’ (9 genes), ‘cell morphology’ (19 genes), and ‘nervous system development and function’ (11 genes). Similarly, 76 IPA functions were affected in LR rats with ‘cellular assembly and organization’ (73 genes), ‘cellular function and maintenance’ (77 genes), ‘cell-to-cell signaling and interaction’ (53 genes), ‘nervous system development and function’ (83 genes), ‘cell morphology’ (74 genes), and ‘cellular development’ (30 genes) being among the functions overrepresented. It is noteworthy that both the GO and IPA analyses confirmed that FST exposure affected the transcription of genes related to hippocampal cellular proliferation in both novelty-seeking phenotypes.

Zooming in the specific molecular pathways associated with the observed gene expression changes, data mining through IPA exposed markedly divergent mechanisms in the hippocampus of the two novelty-seeking phenotypes in response to FST stress. Overall, 49 canonical IPA pathways comprising 1 to 11 genes each were depicted as significantly changed following FST in HR rats, versus 62 pathways comprising 1 to 16 genes each in LR rats. Only 15 pathways were common in both HR and LR rats, and these were largely represented by different genes due to the low number of common genes affected in both novelty-seeking phenotypes. Notably, in HR rats, ‘EIF2 Signaling’ and ‘Axonal Guidance Signaling’ were among the top five pathways affected upon FST exposure, whereas in LR rats, these were ‘Ephrin Receptor Signaling’ , ‘Clathrin-mediated Endocytosis Signaling’ , and ‘ERK/MAPK Signaling’ (Additional file 4).

### Mammalian Adult Neurogenesis Gene Ontology (MANGO) analysis

In order to decipher the impact of FST exposure on the regulation of neurogenesis-related mechanisms, the MANGO was implemented [19]. In particular, the MANGO database was screened for genes that were statistically significantly changed in our data. According to this form of analysis, FST exposure induced a phenotype-dependent expression of neurogenesis-related transcripts in the hippocampus of HR and LR rats. In particular, seven neurogenesis-related genes were statistically significantly upregulated in LR (i.e., *Ephb2*, *Nog*, *Ntf3*, *Tgfb1*, *Smad7*, *Sox2*, and *Srr*), as compared to only three genes in HR rats (i.e., *Bmpr1a*, *Gsk3b*, and *Jag1*). This analysis revealed that exposure to the same FST regimen induced a phenotype-dependent regulation of unique neurogenesis-related genes in the hippocampus of the two novelty-seeking phenotypes.

### IPA network analysis

**Figure 4:**
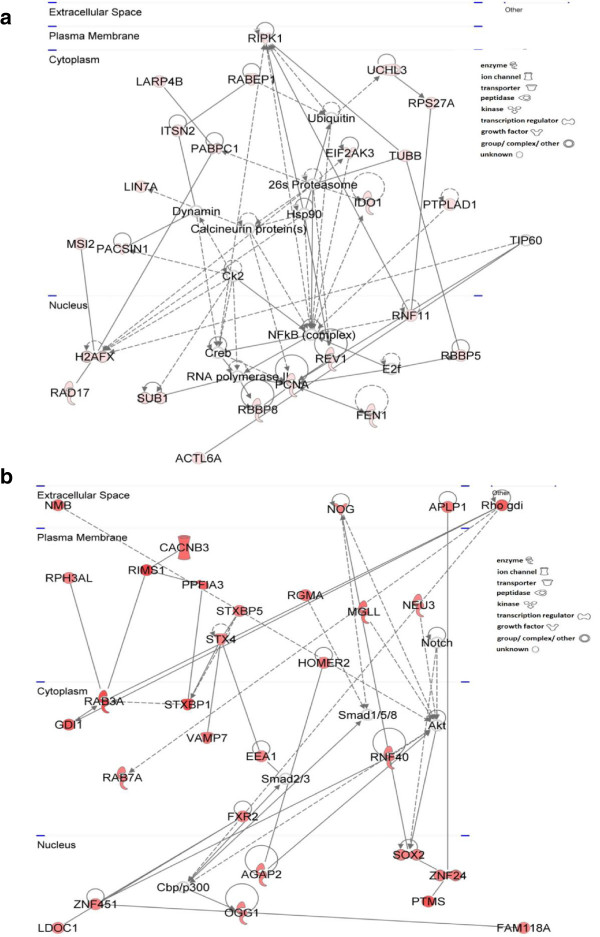
**Top Ingenuity networks of genes whose expression was significantly altered in the hippocampus upon FST exposure. (a)** HR (24 upregulated transcripts) and **(b)** LR (30 upregulated transcripts) rats. Upregulated molecules are shown in *red* and increasing color intensity reflects higher fold changes. Genes with baseline expression are shown in *white. Arrows* point to the activated molecule in a signaling pathway or the product of a metabolic pathway. *Solid lines* indicate direct and *dashed lines* indirect interactions. *Geometrical shapes* (nodes) reflect the key functions of the specific protein.

Finally, network analysis was performed to unravel the intricate relationships between the different significantly changed pathways. In HR rats, 13 networks were generated by IPA versus 25 in LR rats. Consistently with all other levels of analysis, the molecular networks that emerged for the HR and LR responses to FST exposure were markedly different. This indicates that not only the individual genes and pathways are characteristic of each of the two behavioral phenotypes, but also the cascades of molecular events (i.e., the networks of genes from different pathways) giving rise to the depressive-like phenotype are distinct. The top networks of genes whose expression was significantly altered in the hippocampus of the two novelty-seeking phenotypes are presented in Figure 4.

## Discussion

Herein, we report for the first time the marked divergence of the FST-induced hippocampal gene expression signatures between HR and LR rats, despite their comparable depressive-like status during the FST. These molecular differences are reflected in both the extent of transcriptional remodeling (number of significantly changed genes) and the types of molecular pathways affected following FST exposure. A markedly higher number of genes (i.e., 2.28-fold) were statistically significantly changed following FST in LR as compared to their HR counterparts (i.e., 589 versus 258), while only a strikingly low number of genes (i.e., 20 genes) were commonly altered in both novelty-seeking phenotypes. These distinct gene expression signatures support the notion that the hippocampus of HR and LR rats undergoes distinct transcriptional remodeling in response to the same environmentally induced stress, thus yielding a different FST-related endophenotype, despite the seemingly similar depressive-like phenotype in this test.

### Structure of HR and LR rats' behavior in the FST

HR and LR rats present a distinct behavioral pattern in the pre-test session but develop comparable depressive-like status in the second FST session. Notably, LR rats spent less time swimming around the cylinder during the pre-test session, as compared to their HR counterparts. Present behavioral findings essentially replicate previous results from our lab; Antoniou et al. [4] reported that HR and LR rats develop a different behavioral FST pattern characterized by a common behavioral state of ‘despair’ , but distinct active behavioral reactions are expressed during the first exposure to water [4]. The structure of HR and LR rats' behavior during the second FST session is similar between the two novelty-seeking phenotypes, as shown herein and as previously reported [4,12,13]. The majority of studies in this field typically quantify only the second FST session, as it is relevant to the screening of antidepressant drugs [12]. However, it is noteworthy that the pre-test session reflects rats' responsiveness to the presentation of a novel swim stressor that may ultimately lead to the modulation of behavior during subsequent exposure to the stressful stimulus (e.g., induction of learned helplessness) or even to the differential regulation of relevant endophenotypes (i.e., neurobiological indices) without apparent effect in the behaviors assessed [12].

### FST induces differential regulation of neurogenesis-related transcripts in HR compared to LR

In the present study, both the GO and IPA analyses demonstrate that FST exposure affected the transcription of genes related to hippocampal cellular proliferation and homeostasis in both novelty-seeking phenotypes. According to the MANGO analysis, FST exposure induced a phenotype-dependent expression of neurogenesis-related genes in the hippocampus of HR and LR rats. In particular, only three neurogenesis-related genes were statistically significantly upregulated in HR (i.e., *Bmpr1a*, *Gsk3b*, and *Jag1*), as compared to seven genes in LR rats (i.e., *Ephb2*, *Nog*, *Ntf3*, *Tgfb1*, *Smad7*, *Sox2*, and *Srr*). Importantly, exposure of HR rats to the FST paradigm induced hippocampal *Gsk3b* (glycogen synthase kinase 3 beta; +2.089) mRNA levels. Gsk3b is a negative regulator of the b-catenin and the canonical Wnt signaling pathway, which modulate hippocampal neurogenesis [20,21], but has also been reported to modulate Notch signaling by enhancing its stability [22]. Notably, overexpression of *Gsk3b* in the murine hippocampus causes dramatic alterations in both the dendritic tree morphology and the postsynaptic densities of newborn neurons and has been proposed as a target for the treatment of Alzheimer's disease [23]. Moreover, FST exposure in HR rats upregulated *Bmpr1a* (bone morphogenetic protein receptor, type IA; +2.275) and *Jag1* (jagged; +2.163; the ligand of Notch1), which have been reported to affect neurogenesis in the adult hippocampus either directly or indirectly [24,25].

In LR rats, the FST induced the transcriptional activation of genes that positively regulate neurogenesis in the adult hippocampus. Notably, *Ntf3* (neurotrophin 3; +2.469) facilitates hippocampal plasticity by promoting neurogenesis in the dentate gyrus [26], *Nog* (noggin; +2.177) is a BMP antagonist that diverts stem cells from a glial to a neuronal fate [27], and *Sox2* (SRY (sex-determining region Y)-box 2; +2.201) maintains neural stem cell properties, including proliferation/survival, self-renewal, and neurogenesis [28]. Moreover, FST exposure led to overexpression of *Tgfb1* (transforming growth factor, beta 1; +2.219) and *Smad7* (SMAD family member 7; +2.443) mRNA in the hippocampus of LR rats. Interestingly, TGFb1 has been recognized as a negative regulator of adult neurogenesis since infusion of TGFb1 into the ventricles of the adult rat brain reduced the number of proliferating cells in the hippocampus [29], while SMAD7 may either directly antagonize TGF receptor signaling [30] or act in a TGF receptor-independent manner and further suppress neurogenesis [31]. Although not registered in the MANGO database, several fibroblast growth factor (FGF)-related genes were upregulated in the hippocampus of LR rats following FST, namely *Fgf12* (fibroblast growth factor 12; +3.116), *Fgf13* (fibroblast growth factor 13; +2.465), *Fgfr2* (fibroblast growth factor receptor 2; +2.088), and *Frs3* (fibroblast growth factor receptor substrate 3; +2.113). Intriguingly, upregulation of *Fgfr2* upon FST exposure may mediate the neuroprotective effects of FGF2 on hippocampal formation [32]. Thus, it could be hypothesized that FST has a beneficial effect in the hippocampus of LR rats by promoting neuroprotective processes.

Intriguingly, FST induced phenotype-dependent effects on the transcription of prominent serotonin (5-HT)-related enzymes. In the hippocampus of LR rats, FST upregulated *Tph2* (tryptophan hydroxylase 2; +2.145), the rate-limiting enzyme of 5-HT biosynthesis, that is possibly indicative of serotonergic activation in this brain region. On the other hand, in HR rats, *Ido1* (indoleamine 2,3-dioxygenase; +2.302) levels were induced. It is noteworthy that IDO1 is a proinflammatory enzyme that converts l-tryptophan (the precursor of serotonin; 5-HT) to kynurenine and its neurotoxic metabolites that are able to produce oxidative stress by increasing the production of reactive oxygen species (ROS) or to overstimulate hippocampal *N*-methyl-d-aspartate (NMDA) receptors and lead to apoptosis and hippocampal atrophy [33].

Overall, FST exposure induced a phenotype-dependent modulation of neurogenesis-related genes in the hippocampus of the two novelty-seeking phenotypes. Notably, the hippocampus is a stress- and glucocorticoid (GC)-sensitive brain region. In particular, within the dentate gyrus (DG), there exists a high density of glucocorticoid receptors (GRs) that respond to enhanced circulating GCs [34]. According to previous reports from our group and others, serum corticosterone concentrations are enhanced by FST exposure irrespective of the HR/LR phenotype [12,13] and this could negatively affect hippocampal cell proliferation [35]. Moreover, it has been shown that hippocampal DNA damage occurs immediately after exposure of male Wistar rats to FST [36] and that exposure of female rats to the same regimen may reduce cell survival in the hippocampus, probably by increasing corticosterone levels [37]. Indeed, this profile nicely fits in with the transcriptomic remodeling observed in HR rats in response to FST in the current study. On the other hand, recent data also support a beneficial role for acute stress on the hippocampus. In particular, exposure of rats to restraint stress increased cell proliferation and astrocytic fibroblast growth factor 2 (*fgf2*) expression in the dorsal hippocampus [38]. Remarkably, in the present study, exposure of LR rats to FST resulted in enhanced expression of hippocampal *Ffgr2* mRNA levels. Importantly, FST appears to promote neurogenic processes in LR rats. These findings are in accordance with the enhanced neurogenesis observed in the DG of LR rats at baseline. As shown before, cell proliferation in the DG of LR Wistar rats, evaluated by the incorporation of 5-bromo-2′-deoxyuridine (Brdu) in progenitors, was twice that observed in their HR counterparts [39]. Similarly, selectively bred HR and LR (bHR/bLR) lines of Sprague-Dawley rats revealed, through Ki67 immunohistochemistry, enhanced cell proliferation in the DG of developing bLR versus bHR pups [40]. In conclusion, the increased rates of hippocampal neurogenesis in LR rats at baseline appear to be maintained in response to FST-induced stress, with the activation of numerous relevant processes. These molecular changes contribute to the markedly different molecular *milieu* of LR compared to HR rats that may predispose to distinct long-term behavioral or pharmacological responses.

### FST induced upregulation of neuroplasticity-related transcripts in LR rats

**Figure 5:**
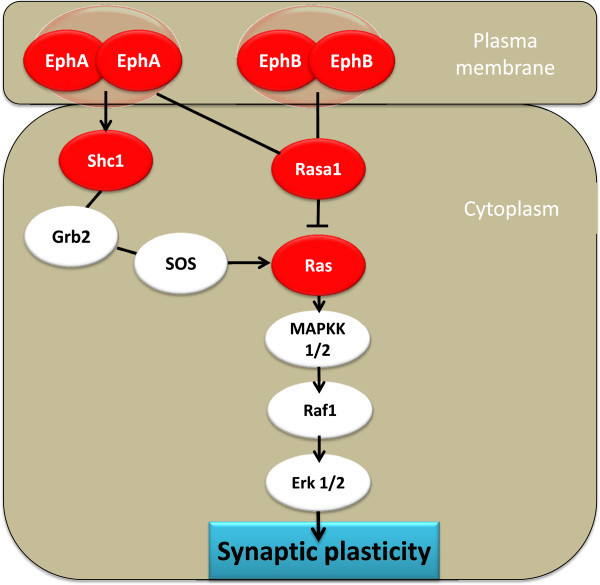
**‘Ephrin Receptor Signaling’ was the second canonical IPA pathway induced in LR rats upon FST exposure.** Upregulated molecules are shown in *red. Blind lines* denote binding, *arrows* signify activation, whereas *T-like lines* indicate inhibition. *EphA5* Ephrin A5 receptor, *EphB2* Ephrin B2 receptor, *Erk1/2* mitogen-activated protein kinase 1, *GRB2* growth factor receptor-bound protein 2, *MAPKK1/2* mitogen-activated protein kinase 2, *RASA1* RAS p21 protein activator (GTPase-activating protein) 1, *RAF1* v-raf-1 murine leukemia viral oncogene homolog 1, *Shc1* Src homology 2 domain containing) transforming protein 1, *SOS* son of sevenless homolog 2.

Neuroplasticity refers to the brain's ability to change its structure and function during maturation, learning, environmental challenges, pathology, and stress. Synaptic pruning and remodeling of the postsynaptic cytoskeleton comprise structural changes that are possible in the adult brain. Interestingly, exposure to FST upregulated genes directly or indirectly involved in neuroplasticity in a phenotype-specific manner in LR rats. Ephrins and their receptors govern the topographic guidance of axons during central nervous system (CNS) development but are also implicated in neuronal plasticity in the adult brain [41]. Herein, the “Ephrin Receptor Signaling” pathway was the second among the top five canonical IPA pathways affected in the hippocampus of LR rats upon FST exposure (Figure 5). Specifically, FST resulted in the upregulation of both *Ephb2* (+2.167) and *Epha5* (+2.879) transcripts. Ephrin receptors are the largest known family of receptor tyrosine kinases in the mammalian genome and are divided into A and B subclasses based on affinity for their membrane-associated ligands, ephrin-As and ephrin-Bs. Notably, *Ephα5* is expressed at both the protein and mRNA levels in the adult murine hippocampus where it has been hypothesized to promote synaptic plasticity [42]. Most importantly, *Ephb2* is expressed in the adult hippocampus and regulates, among others, synaptic structure, NMDA receptor clustering and function, and LTP [43].

In line with this evidence, the IPA functions predicted to be statistically significantly enhanced in LR rats upon FST exposure involved ‘growth of neurites’ (*z*-score 2.825), ‘LTP of synapse’ (*z*-score 2.396), ‘microtubule dynamics’ (*z*-score 2.453), ‘organization of cytoplasm’ (*z*-score 2.276), and ‘organization of cytoskeleton’ (*z*-score 2.269), all of which are directly related with neuroplasticity. Of particular interest was the upregulation of a number of neuroplasticity-related synaptic players in the hippocampus of LR rats, such as *Ddn* (dendrin; +2.225), a postsynaptic regulator of the structure of synaptic cytoskeleton, and *Stx4* (syntaxin 4; +2.178), an essential postsynaptic component for synaptic plasticity in hippocampal neurons [44]. Moreover, the upregulation of both *Pfn* (profilin) isoforms (i.e., 1 and 2; +2.709 and 2.396, respectively) that are prominent regulators of actin dynamics in the CNS is indicative of the cytoskeletal reorganization that possibly takes place upon FST exposure in the hippocampus of LR rats [45]. Moreover, FST appears to stimulate neurotransmitters' release in LR rats, as reflected by the upregulation of *Dnm1* (dynamin 1; +2.388) which is involved in exo-endocytosis of synaptic vesicles [46] and *Syt12* (synaptotagmin 12; +2.352) which is a synaptic vesicle phosphoprotein that modulates neurotransmitter release [47]. Hippocampal synaptotagmin mRNA levels were found to be enhanced upon immobilization stress in rats, in view of the effects of stress on synaptic plasticity [48]. Remarkably, exposure of LR rats to FST resulted in the upregulation of genes implicated in core neuroadaptive processes in the adult hippocampus.

### FST induced apoptosis-related mechanisms in the hippocampus of HR rats

**Figure 6:**
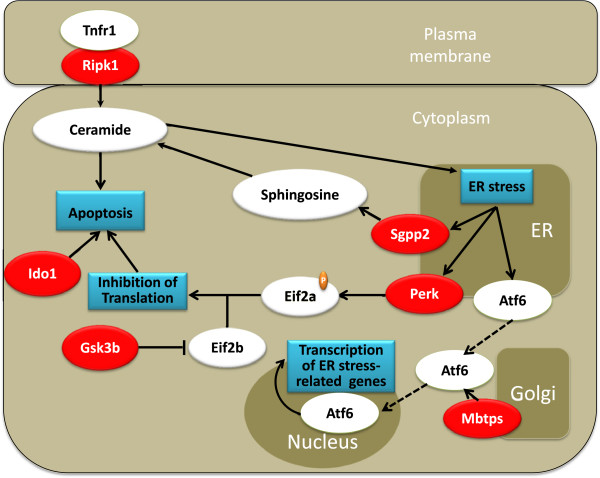
**ER stress and eIF2a pathways appear to be induced in the hippocampus of HR rats.** Induction of these pathways could be implicated in the mobilization of apoptotic mechanisms in hippocampal neurons of HR rats upon FST exposure. Upregulated genes are shown in *red. Blind lines* denote binding, *arrows* signify activation, whereas *T-like lines* indicate inhibition. *ATF-6* activation transcription factor 6, *eIF2a* eukaryotic initiation factor 2a, *eIF2b* eukaryotic initiation factor 2b, *Gsk3b* glycogen synthase kinase-3 beta, *Ido1* indoleamino-2,3-dioxygenase, *MBTPS* membrane-bound transcription factor peptidase, *Perk* eIF2a kinase 3, *Sgpp2* sphingosine-1-phosphate (S1P) phosphatase 2, *Ripk1* TNF receptor (TNFRSF)-interacting serine-threonine kinase 1, *TNFR1* tumor necrosis factor receptor superfamily.

The top canonical IPA pathway affected in HR rats upon FST exposure was that of eIF2 (eukaryotic translation initiation factor 2) signaling. The eIF2 complex is essential in all eukaryotes for protein synthesis, since it recruits the initiator methionyl tRNA (Met-tRNA) to ribosomes to begin translation. In addition, multiple endoplasmic reticulum (ER) stress-related genes were overexpressed in the hippocampus of HR rats following FST exposure, including *Perk* (*eif2ak3*; eIF2a kinase 3; +2.053), *Sgpp2* (sphingosine-1-phosphate (S1P) phosphatase 2; +3.121), and *Mbtps2* (membrane-bound transcription factor peptidase 2; +2.593). As depicted in Figure 6, *Perk* upregulation could lead to phosphorylation-induced inhibition of eIF2α that could in turn result in global reduction of protein translation. In addition, upregulation of *Gsk3b* (+2.089) may inhibit the activity of eIF2b (eukaryotic initiation factor 2B) that could also lead to inhibition of protein translation in neuronal cells [49]. Interestingly, it has been proposed that inhibition of protein translation is a mode of inducing neuronal apoptosis and neurodegeneration in Alzheimer's disease [50,51]. Notably, induction of *Mbtps2* that encodes an intramembrane zinc metalloprotease involved in ER stress response may enhance the activation of target genes related to the ER stress pathway through an *Atf-6* (activating transcription factor 6)-dependent mechanism. In addition, *Sgpp2* is an enzyme localized in cell and ER membranes that forms sphingosine from S1P that in turn is N-acylated to ceramide; both ceramide and sphingosine have been associated with induction of ER stress, growth arrest, and apoptosis in mammalian cells [52-54]. FST also upregulated *Rip1* (TNF receptor (TNFRSF)-interacting serine-threonine kinase 1; +2.249), a kinase originally discovered via its interaction with the death domain of TNFR1 [55]. Importantly, *Rip1* is a central initiator of cell death and may also play a role in the ceramide death pathway [51]. Overall, it appears that in HR rats FST induced the overexpression of multiple genes directly and indirectly associated with apoptotic mechanisms in the adult hippocampus.

## Conclusions

Herein, we report that FST-induced hippocampal gene expression signatures between HR and LR rats are markedly divergent. The two novelty-seeking phenotypes present a distinct behavioral pattern in the pre-test session but develop comparable depressive-like status in the second session of the FST. At 24 h post-FST exposure, hippocampal gene expression signatures between HR and LR rats were markedly divergent, despite their similar FST performance. These molecular differences are reflected in both the extent of transcriptional remodeling (i.e., number of significantly changed genes) and the types of molecular pathways affected following FST exposure. A markedly higher number of genes (i.e., 2.28-fold) were statistically significantly changed following FST in LR rats, as compared to their HR counterparts. Specifically, global transcriptomic alterations suggest that following FST exposure, neurogenesis and neuroplasticity-related processes are enhanced in LR rats, while pathways that are directly or indirectly implicated in apoptotic cascades are induced in their HR counterparts. Importantly, the present data extend previous findings on the differential modulation of neurogenesis between the two novelty-seeking phenotypes at baseline.

Overall, the present data suggest a complex pattern of molecular modifications that likely reflect distinct neurobiological processes occurring between the adult HR and LR hippocampus upon FST exposure. Strikingly, susceptibility to stress is individually different. Gaining an insight into individual differences may elucidate the neurobiological substrate for this vulnerability, help clarify pathophysiological mechanisms, and hopefully aid in developing strategies to discover more effective pharmacotherapies [2].

## Methods

### Animals

Male *Sprague-Dawley* rats (*N* = 24) were purchased from Athens' Pasteur Institute. Rats aged 80–90 days old and weighed 250–300 g at the beginning of the experiments. Rats were group housed in plastic cages measuring 570 mm × 380 mm × 200 mm, with food pellets and tap water available *ad libitum*, under controlled laboratory conditions (i.e., 12-h light/dark with lights on at 0700 hours and a constant temperature of 21°C ± 1°C), as previously described [12]. Approval to conduct the experiments described has been obtained from the animal subjects review board of our institution. All animal experiments have been carried out in accordance with the European Communities Council Directive of 24 November 1986 (86/609/EEC). Efforts were made to minimize the numbers of animals used and to reduce their suffering.

### Classification into HR/LR groups

All rats were classified as HR and LR according to their frequency of rearing (counts registered when the rat's body inclined vertically with hind paws on the floor and forepaws on the wall of the cage) in an activity chamber, as in previous studies [4,12,56,57]. Vertical activity has been used, apart from general locomotion, as a sole criterion for assignment of rats into groups during their exposure to novelty [58,59]. The rats were ranked using the frequency of vertical counts (rearing). Animals above the median were designated as HR, while the rest were classified as LR. Testing was conducted in two clear Plexiglas activity chambers measuring 430 × 430 × 300 mm (Med Associates Inc., St Albans, VT, USA), and behavior was recorded for a 15-min observation period, as previously described [12,56].

### Forced swim test (FST)

All rats were acclimatized to the test room for 1 h before the beginning of the experiment. HR and LR rats (*N* = 7 per phenotype) were individually placed in a cylindrical tank measuring 50 cm in height and 20 cm in width or were left undisturbed in their home cage and served as controls (*N* = 5 per phenotype). The tank was filled with water (24°C ± 1°C), and water was changed after each session. The animals were forced to swim for a 15-min period (pre-test) and 24 h later were subjected to a 5-min swimming session (test) [60]. The total duration of floating (immobility), swimming, and climbing periods were scored by the same observer for the first 5 min of each session (pre-test and test), as mentioned before [12,61]. Rats were considered to show immobility when they floated without struggling, making only those movements necessary to keep their heads above the water. Swimming was recorded when they actively swam around in circles, while climbing was scored when the rats climbed the walls of the cylinder. Following swimming sessions, the rats were removed from the tank, carefully dried in heated cages, and then returned to their home cages. At 24 h following the second session of the FST, all HR and LR rats (stressed and respective controls) were sacrificed by rapid decapitation with the aid of a guillotine. Notably, in earlier studies, we and others have chosen this wait period in order to examine prolonged stress-induced effects on gene expression alterations within a short time period following the stress session [17,61-63].

### RNA isolation and qualitative and quantitative analyses

Following decapitation, the brain was removed from the skull and the region of the hippocampus was rapidly isolated on ice. Sample preparation, hybridization, washing, and staining were performed using the direct hybridization gene expression assay by Illumina, involving a first- and second-strand reverse transcription step, followed by a single *in vitro* transcription (IVT) amplification that incorporates biotin-labeled nucleotides, array hybridization, washing, blocking, and streptavadin-Cy3 staining. Hippocampal samples (*N* = 5 per group per phenotype) were analyzed independently on whole rat genome Illumina arrays (RatRef-12 Expression BeadChip), containing 22,523 probes selected primarily from the NCBI RefSeq database (Release 16), representing approximately 22,260 coding transcripts. The arrays were scanned using the BeadArray Reader (Illumina, San Diego, CA, USA).

### Statistics, bioinformatic analysis, and data mining

Statistical analysis of behavioral data was performed with SPSS version 20 statistical software (SPSS Inc., Chicago, IL, USA). Results were analyzed using one-way analysis of variance (ANOVA) for the factor of phenotype (HR versus LR). Specifically, separate one-way ANOVAs were performed for each FST session. All data were first tested against ANOVA data assumptions.

The resulting gene expression data sets from the 20 hippocampal samples were scanned using the Illumina GenomeStudio Gene Expression Module (version 2010.2) at default specifications and thresholds. The scanned microarray image files were pre-processed using quantile normalization without background correction [64]. Bioinformatic analysis was performed using the BRB-Array Tools Version 4.2.1 [65]. All intensity values were transformed to the log2 basis. Differentially expressed genes were then identified using a random-variance *t* test, with a *p* value threshold of ≤0.01. To increase stringency, only genes with ≥2-fold change were further considered for the purposes of this study. Significantly changed genes were annotated according to the Gene Ontology classification system into ‘biological process’ (BP), ‘molecular function’ (MF), and ‘cellular component’ (CC) by using the GeneCodis3 software, a web tool for functional interpretation of experimental data. The application is freely available at http://genecodis.cnb.csic.es[66-68]. In particular, unique gene symbols (ENTREZ) corresponding to HR and LR data sets, respectively, were processed in the application ‘Comparative analysis’ of GeneCodis3 for *Rattus norvegicus*, the GO level (level 5), and the statistical parameters of the analysis. The submission of two different lists at the same time results in simultaneous modular and singular enrichment analyses for each one. Hypergeometric distribution was used for the calculation of *p* values and the FDR method to correct *p* values for multiple hypothesis testing. Statistical significance for both tests was determined at the *p* < 0.05 level. Annotations were then retrieved for each data set in order for unique and common genes to be further analyzed.

Literature mining and biological interpretation of significantly changed genes was performed with the Ingenuity Pathway Analysis software (IPA, Ingenuity Systems, http://www.ingenuity.com). Data of analysis are experimentally observed and come from Ingenuity Knowledge Base. Fisher's exact test is used for the grouping of analyzed genes into biofunctions and canonical pathways by calculating the *p* value of a given category. Statistical significance association of genes to the functional or pathway categories is considered for *p* values less than 0.05 and is expressed as the negative log of the *p* value. Additionally, the activation state of a functional category is predicted by calculating the *z*-score. A predicted increase in activation of a function is considered when the *z*-score is ≥2, and accordingly, a predicted decrease is considered when the *z*-score is ≤2.

In order to decipher the impact of FST exposure on the regulation of neurogenesis-related mechanisms, the Mammalian Adult Neurogenesis Gene Ontology (MANGO) was further implemented [19]. The MANGO accounts for a database of genes mapped to cell types and processes that have been curated from the literature concerning adult neurogenesis [19]. Herein, the MANGO database was screened for genes that were statistically significantly changed in our data sets.

## Competing interests

The authors declare that they have no competing interests.

## Authors’ contributions

PMP, DS, PAT, and ZPD conceived the study and participated in its design and coordination, the interpretation of results, and the drafting of the manuscript. MP, DN, and MK performed the bioinfomatic analysis of the microarray data. CRT participated in the generation of the microarray data. All authors read and approved the final draft.

## Supplementary Material

Additional file 1 **HR datasheet.** Statistically significantly changed transcripts between FST-exposed and control (unstressed) HR rats. Click here for file

Additional file 2 **LR datasheet.** Statistically significantly changed transcripts between FST-exposed and control (unstressed) LR rats. Click here for file

Additional file 3 **Common transcripts between HR and LR.** Statistically significantly changed transcripts that were similarly affected in HR and LR rats in response to FST. Click here for file

Additional file 4 **IPA pathways.** Top-5 of statistically significantly changed IPA pathways in HR and LR rats in response to FST. Click here for file
